# Turbulence induced shear controllable synthesis of nano FePO_4_ irregularly-shaped particles in a counter impinging jet flow T-junction reactor assisted by ultrasound irradiation

**DOI:** 10.1016/j.ultsonch.2023.106590

**Published:** 2023-09-08

**Authors:** Bin Dong, Yanqing Guo, Jie Yang, Xiaogang Yang, LuLu Wang, Dechun Huang

**Affiliations:** a Department of Mechanical, Materials and Manufacturing Engineering, The University of Nottingham Ningbo China, University Park, Ningbo 315100, PR China; b Department of Pharmaceutical Engineering, China Pharmaceutical University, Nanjing 210009, PR China; c Engineering Research Center for Smart Pharmaceutical Manufacturing Technologies, Ministry of Education, China Pharmaceutical University, Nanjing 210009, PR China; d School of Natural Sciences, University of Hull, Hull HU6 7RX, UK

**Keywords:** Turbulence shear, T-junction impinging streams, Ultrasonic irradiation, Micromixing, CFD modelling, FePO_4_ (FP) nanoparticles

## Abstract

FePO_4_ (FP) particles with a mesoporous structure amalgamated by nanoscale primary crystals were controllably prepared using an ultrasound-intensified turbulence T-junction microreactor (UTISR). The use of this type of reaction system can effectively enhance the micro-mixing and remarkably improve the mass transfer and chemical reaction rates. Consequently, the synergistic effects of the impinging streams and ultrasonic irradiation on the formation of mesoporous structure of FP nanoparticles have been systematically investigated through experimental validation and CFD simulation. The results revealed that the FP particles with a mesoporous structure can be well synthesised by precisely controlling the operation parameters by applying ultrasound irradiation with the input power in the range of 0–900 W and the impinging stream volumetric flow rate in the range of 17.15–257.22 mL·min^−1^. The findings obtained from the experimental observation and CFD modelling has clearly indicated that there exists a strong correlation between the particle size, morphology, and the local turbulence shear. The application of ultrasonic irradiation can effectively intensify the local turbulence shear in the reactor even at low Reynolds number based on the impinging stream diameter (Re < 2000), leading to an effective reduction in the particle size (from 273.48 to 56.1 nm) and an increase in the specific surface area (from 21.97 to 114.97 m^2^·g^−1^) of FP samples. The FP irregularly-shaped particles prepared by UTISR exhibited a mesoporous structure with a particle size of 56.10 nm, a specific surface area of 114.97 m^2^·g^−1^ and a total pore adsorption volume of 0.570 cm^3^·g^−1^ when the volumetric flow rate and ultrasound power are 85.74 mL·min^−1^ and 600 W, respectively.

## Nomenclature

**Abbreviations**
TISR: T-type impinging stream intensified microreactor
UTISR: Ultrasound-intensified turbulence T-junction microreactor
FP: Iron phosphate
Re: Reynolds number
*Q*: Volumetric feeding rate, mL/min
*C*: Reagent concentration, mol/L
*V_TISR_*: Volume of mixing zone in TISR, cm^3^
*t_R_*: Mean residence time, s
*t_M_*: Micromixing time, s
*t_N_*: Nucleation time, s
*v*: Velocity of the impinging stream solutions, m/s
*q_TISR_*: Discharge ratio, dimensionless
*A*: Cross-sectional area, m^2^
*L*: Length, mm
*d*: Tube diameter, m
*P*: Input power, W
*P_IS_*: Input power of impinging stream, W
*P_UI_*: Input power of ultrasonic irradiation, W
*r_o_*: Overall reaction rate
*k_l_*: Mass transfer coefficient
*C_o_*: Reagent initial concentration, mol/L
d*_32_*: Mean diameter over the surface distribution
*V_l_*: Liquid volume, cm^3^
*V_T_*: Volume of reactor chamber, cm^3^
*m_p_*: Mass of the solid particles, kg
*F_c_*: Shape factor
*d*_43_: Mean diameter over the volume distribution

**(Herdan or De Brouckere diameter)**
*P_a_*: Amplitude of the sound pressure, Pa
*f*: Frequency, Hz
*I*: Sound intensity, W/m^2^
*P_UI_*: Ultrasonic power, W
*A*: Tip area of the ultrasonic transducer, m^2^
*ΔP*: Pressure drop
*D_eddy_*: Eddy diffusivity of the reactant solutions
*v_light_*: Sound speed in the water, m/s

**Greek letters**
*φ*: Ratio of the cross-sectional areas, dimensionless
*α*: Ratio of the volumetric flow rate, dimensionless
*ε*: Micromixing turbulent energy dissipation rate, J/s·kg
*ξ_T_*: Local loss coefficient
*λ*: Kolmogorov length
*ρ_ms_*: Densities of the mixed solution, kg/m^3^
*μ_ms_*: Kinematic viscosity of the mixed solution, Pa·s
*ρ_p_*: Density of the solid particles, kg/m^3^
*μ_l_*: Viscosity of the liquid, Pa·s
*ρ_l_*: Density of the liquid, kg/m^3^

## 1 Introduction

For the synthesis processes of various functional micro/nano particles using chemical reactors such as mixing tanks, the hydrodynamic mixing effect plays a crucial role in adjusting the energy, heat and mass transfers in the process, consequently affecting the properties of final products [1], [2], [3]. At molecular scale, the selectivity of chemical reaction is significantly influenced by the mixing process, in particular the micromixing time, which is to a great extent affected by both the turbulent eddies characterized by the macromixing and mesomixing [4], [5]. T-type impinging stream microreactor (TISR) is the simplest mixing device which can provide extremely rapid micromixing and effectively enhance the process turbulence intensification, delivering homogeneous supersaturation level before particle nucleation process and leading to the final particle products with relatively uniform properties [6], [7]. Generally, the TISR consists of two inlet small size tubes and one outlet tube to form the main flow channel configuration with a T-shape. The liquid/gas streams with high velocity from both inlet tubes impinge upon each other in a small mixing chamber to generate strong mixing effects, usually in a highly turbulent status such that intensive micromixing can occur. This results in the reactants becoming homogeneously distributed in an extremely short period of time, whichenables the mass transfer rate, energy dissipation rate, and nucleation rate to be enhanced effectively [8], [9]. The liquid impinging stream can also change the kinetic energy and redistribution of the molecules of the reactants by inducing local pressure fluctuation [10]. Previous studies have demonstrated that by using a confined impinging jet device, the control of the ratio of the assembly time and micromixing time can even significantly influence the pDNA payload, composition, and size of plasmid DNA/polycation complex nanoparticles [11]. However, the rapid generation of solid products and/or by-products may result in irreversible clogging of microchannels, which is one of the biggest disadvantages for using the TISR [12]. Several methods have been applied to prevent the contact of as-synthesised solids on reactor walls, including the use of segmented liquid flows [13], [14], [15]. In addition, the efficiency of chemical reactions may be reduced as the result of the existence of additional solvent in the system, which may be incompatible with the reagents and detrimental to the main reactions [16].

Particle synthesis with the addition of ultrasound intensification has been applied in the industrial manufacturing process and particle material synthesis due to its cost-effective, high efficiency and environmental-friendly nature [17], [18]. Ultrasonic-intensification assisted reactors can be characterised by two types, the former being probe-horn reactors (including vertical probe horn and longitudinal horn) and the latter being cup-horn reactors (ultrasonic bath) [19]. Compared with a cup-horn reactor, probe-horn reactors require less energy consumption during the synthesis process [20]. When the liquid medium is irradiated by ultrasound, ultrasonic waves can generate a large number of cavitation microbubbles by inducing acoustic cavitation. As the instantaneous collapse of such cavitation microbubbles in the reactor can cause an extremely high build-up of pressure (up to 1000 atm) and temperature (up to 5000 K) together with the heating and cooling rate (greater than 1010 K·s^-1^) inside the cavitation zone, the chemical and physical changes in the solution are significantly enhanced by the imploding of cavitation microbubbles and induced turbulence [21], [22]. Consequently, the application of ultrasound irradiation can effectively assist in preventing agglomeration among particles and interaction between particles and the reactor surface [23]. Therefore, the adoption of ultrasonic-irradiation is a promising technology that can enhance micromixing, but at the same time overcomes the problem of clogging and slurry transport difficulties in ISR [24], [25], [26].

Iron (III) phosphate (FePO4 or FP) is a promising substance used as fertilizer [27], catalyst [28], [29], [30], and electrode precursor for lithium ion batteries [31]. Previous studies have reported that yolk-shell FePO4 nanospheres with mesoporous nanoyolks were controllably prepared by employing a carbon acidification templating strategy [32]. As an energy storage material, the small particle size (∼150 nm), large surface area (181.3 m2·g^−1^) and mesoporous structure of FP can effectively improve the sodium storage properties. Due to trienzyme-like activities, FP nanoparticles (200–350 nm) prepared by hydrothermal growth method were modified with L-cysteine and revealed excellent treatment of bacterial infections [33]. As a catalyst, trigonal FP nanoparticles (30–60 nm) synthesised by sol–gel method showed high catalytic performance when directly transfering CH4 into HCHO [34]. Therefore, particle morphology optimisation, including fabricating FP with nanostructure and integrated porosity, can ensure effective ion permeation and significantly increase the performance of FP [35]. We have attempted to use an ultrasound-intensified turbulence T-junction microreactor (UTISR) to produce FP nanoparticles in the previous study [36]. By using such a reactor, the synthesised FP particle samples exhibited improved electrochemical performance due to a shortened Li+ diffusion path and also improved the kinetics of the Li+ insertion/extraction process. To the best of our knowledge, only a few studies have reported the adoption of UTISR for preparing FP particles. In addition, the effect of the micromixing in such a reactor on the preparation of FP particles is still not clearly understood and requires further investigation.

This work aims to further investigate the mechanism of the synthesis of FePO4 particles using the UTISR technology, in particular the effect of ultrasound irradiation assisted hydrodynamic micromixing on the particle aggregation and breakage for the synthesised particle size control and morphology under the conditions that various operating parameters are studied. While previous studies have sought the effect of ultrasound power on the FePO4 formation process [37], the present study particularly focuses on the synergistic effects of variable solution volumetric flow rates and the ultrasound irradiation intensity on the hydrodynamic micromixing and synthesis and preparation process of FePO4. In this work, section 2 will present the experimental details for materials preparation and characterisation. Section 3 will illustrate the information about mathematical modelling and simulation. Section 4 will analyse the formation mechanism of FePO4 and the correlation between hydrodynamics and chemical reaction based on experimental and simulation results. The conclusions derived from the study is summarised in section 5.

## 2 Experimental

### 2.1 Sample synthesis

**Fig. 1:**
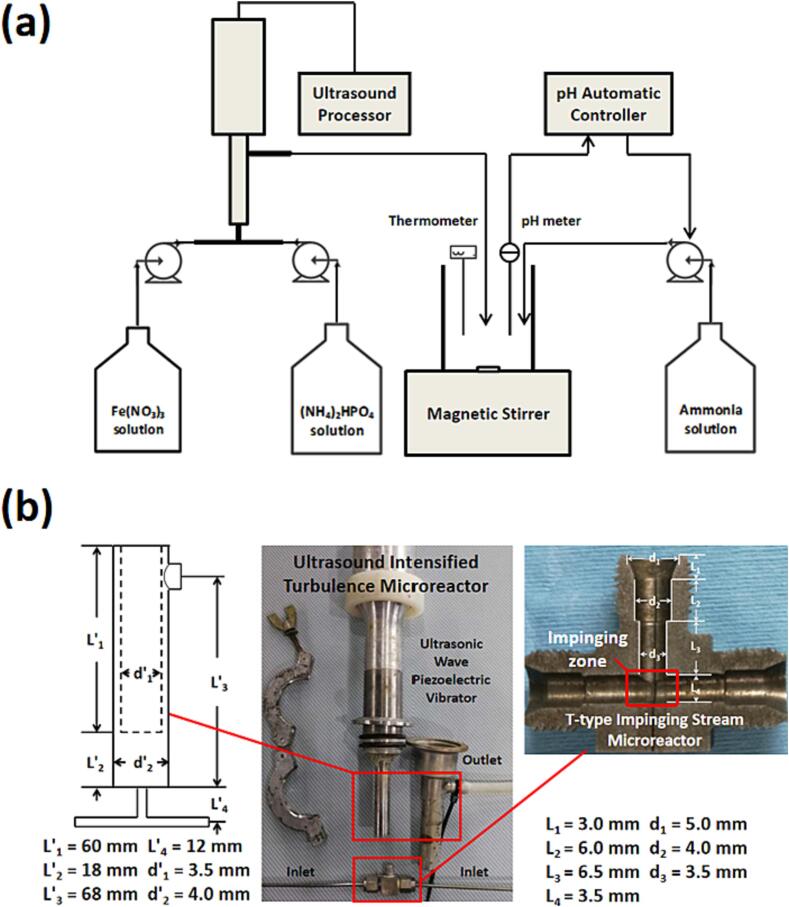
(a) Scheme of experimental set-up and (b) internal structure of TISR and UTISR.

To study the effects of the ultrasound irradiation intensity applied and volumetric feeding rate, two methods were designed for the synthesis of FP nanoparticles. The first type of FP sample was synthesized via an ultrasound-intensified turbulence T-junction microreactor (UTISR). Iron nitrate (Fe(NO3)3, C = 1.0 mol·L^−1^) solution and diammonium phosphate solution ((NH4)2HPO4, C = 1.0 mol·L^−1^) were injected continuously into the two T-type impinging streams of the micromixer (T-mixer), as shown in Fig. 1a. The feeding was realised using two peristaltic pumps (BT100FJ, Baoding Chuangrui, China) to precisely control the volumetric feeding rate (Q) ranging from 17.15 to 257.22 mL·min^−1^. Meanwhile, the outlet of T-mixer was connected with a FS-600pv horn type ultrasonic wave piezoelectric vibrator such that the solution can be irradiated by 20 kHz ultrasound with different intensity (0–900 W). During the synthesis process, the pH value of the solution was kept at 1.70 by adding ammonia solution (1.5 mol·L^−1^) carefully through a pH automatic controller. The obtained FP samples were subsequently washed with deionised water for 3 times, filtrated and then dried in air at 100 °C for 12 h. For comparison purposes, the second type of FP nanoparticles was synthesised using only TISR with a precisely controlled volumetric feeding rate (from 17.15 to 257.22 mL·min-1) when the pH value of the solution was maintained at 1.70. The obtained samples were washed, filtrated and then dried in air at 100 °C for 12 h. The operation parameters of different experiments are shown in Table 1, Table 2, Table 3.

**Table 1:** Investigating the effect of volumetric feeding rate (Group 1).

Samples No.	Ultrasound intensity (W)	Volumetric flow rate of inlet 1 (mL/min)	Volumetric flow rate of inlet 2 (mL/min)
S1	0	17.15	17.15
S2		34.30	34.30
S3		51.44	51.44
S4		68.59	68.59
S5		85.74	85.74
S6		128.61	128.61
S7		171.48	171.48
S8		214.35	214.35
S9		257.22	257.22

**Table 2:** Experiments on the effect of ultrasound intensity (Group 2).

Samples No.	Ultrasound intensity (W)	Volumetric flow rate of inlet 1 (mL/min)	Volumetric flow rate of inlet 2 (mL/min)
S5	0	85.74	85.74
S10	300		
S11	600		
S12	900

**Table 3:** Investigating the synergistic effect of volumetric feeding rate and ultrasound power (Group 3).

Samples No.	Ultrasound intensity (W)	Volumetric flow rate of inlet 1 (mL/min)	Volumetric flow rate of inlet 2 (mL/min)
S11	600	85.74	85.74
S13		68.59	68.59
S14		51.44	51.44
S15		34.30	34.30
S16		17.15	17.15

### 2.2 Characterisation of FP samples

The specific surface area, average primary particle size, and porous distribution were analysed by Brunauer Emmett Teller (BET, Micromeritics ASAP 2020, USA). The particle size distribution was also evaluated by dynamic light scattering (DLS, Anton Paar Litesizer 500, Austria). The crystal structure of samples was analysed by a Bruker D8 series X-ray diffraction using Cu Kα radiation (λ = 1.5406 Å). The scanning range of diffraction angle (2θ) was set 10 ≤ 2θ ≤ 70. The surface morphology of the obtained particles was observed by a scanning electron microscope (Sigma VP ZEISS, Germany).

## 3 CFD modelling of the impinge jet flows in the TISR and UTISR

### 3.1 Mathematical modelling

**Fig. 2:**
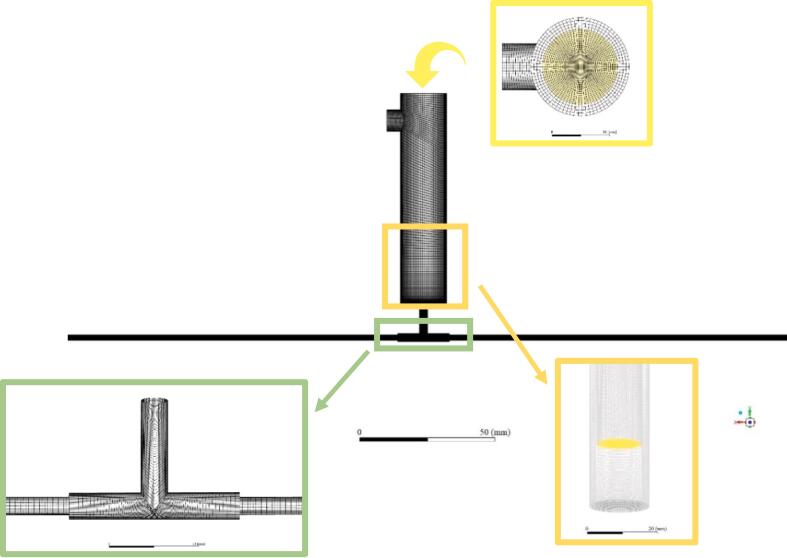
Grid imposed in simulations for the T-junction impinging jet reactor with ultrasound irradiation.

The impinging jet flow together with the flow in the expansion chamber with and without the action of ultrasound was analysed with the assistance of CFD modelling under the conditions that the ultrasound irradiation of the frequency of 20 kHz and power ranging from 0 to 900 W was applied. As the sizes of TISR and UTISR are small, the direct measurement of hydrodynamics such as turbulence becomes difficult, thus the adoption of CFD modelling was used to analyse the intensified effect of ultrasound on this solid–liquid interaction system (both TISR and UTISR). Although the volume fraction of cavitation bubbles induced by ultrasound irradiation is <1%, the cavitation effects cannot be completely ignored, although the ultrasound irradiation is relatively low (∼20kH). The induced lower acoustic pressure enables the initiation of cavitation for porous nanoparticles rather than for solid nanoparticles, which suggests that the presence of pores enhances the sensitivity of the nanoparticles [38]. The size of the majority of acoustic bubbles induced by ultrasound with 20 Hz frequency was estimated within the rough range of 2 × 10–7 to 10–6 m [39]. In this case, the characteristic length scales of the fluid flow are slightly greater than acoustic bubble size, where the Kolmogorov length scale is estimated to the magnitude of 10–6 m by Equation (16). The induced acoustic bubbles were treated as point sources of momentum and energy in the fluid. Therefore, the effect of the bubbles on the fluid flow can be modelled through appropriate boundary conditions (as described in Equation (13)) in the governing equations of the single-phase fluid. To describe the flow in the impinging jet system with a sudden expansion chamber, a model describing the influence of imposition of ultrasound irradiation on the flow dynamics was derived. The assumptions for the derivation are made based on (1) where only the effects of ultrasound irradiation on the liquid bulk flow field are considered; (2) the ultrasound pressure distribution imposed on the flow can be expressed in the form of a sinusoidal pressure wave [12], [22]. It was also assumed that the ultrasound irradiation is acting on the main impinging core zone and the sudden expansion chamber as indicated in Fig. 2. The decay of pressure wave is ignored because of the small reactor volume. The model was coupled with Reynolds-averaged Navier-Stokes equation. As the instantaneous influence of ultrasound irradiation on the flow may be “smoothed out” due to the consequence of time averaging, the time step adopted in the simulations should be smaller than the time scale of the ultrasound irradiation. Here, a time step with 10–7 to 10–8 s may be involved. The distribution of ultrasound irradiation pressure field imposed to the flow is expressed as a function of space and time,

(1) $$p_{\mathrm{up}}=P_{a}cos\left(\omega \left(t-\frac{z}{c}\right)\right)$$

where pup, Pa, ω, z and c are the acoustic pressure amplitude, instantaneous ultrasound pressure, angular velocity and the ultrasound sensor head coordinate in the z direction, respectively. The impact of the imposing ultrasound pressure irradiation on the flow can be modelled as a pressure gradient term. The governing equations employed are thus given by

(2) $$\frac{\partial \rho }{\partial t}+\frac{\partial }{\partial x_{i}}\left(\rho U_{i}\right)=0$$

(3) $$\frac{\partial \rho U_{i}}{\partial t}+U_{j}\frac{\partial \left(\rho U_{i}\right)}{\partial x_{j}}=-\frac{\partial P}{\partial x_{j}}\delta_{\mathrm{ij}}+\frac{\partial }{\partial x_{j}}\left[\mu \left(\frac{\partial U_{i}}{\partial x_{j}}+\frac{\partial U_{j}}{\partial x_{i}}\right)-\rho \overset{¯}{u_{i}^{\prime }u_{j}^{\prime }}\right]+\rho g_{i}+\frac{\partial \overset{¯}{p_{\mathrm{up}}}}{\partial x_{j}}\delta_{\mathrm{ij}}$$

where the stress tensor τ is expressed as:

(4) $$\tau =\mu_{\mathrm{eff}}\left[\mu \left(\frac{\partial U_{i}}{\partial x_{j}}+\frac{\partial U_{j}}{\partial x_{i}}\right)-\rho \overset{¯}{u_{i}^{\prime }u_{j}^{\prime }}\right]$$

here, μeff denotes the effective dynamic viscosity. The instantaneous velocity, stress tensor and ultrasound pressure perturbation can be decomposed into a time-averaged and a fluctuation term, respectively, expressed as:

(5) $$u_{i}=U_{i}+u_{i}^{\prime }p_{\mathrm{up}}=P_{\mathrm{up}}+p_{\mathrm{up}}^{\prime }$$

Coupled with the modified k-ε turbulent model (Equations (8) and (9)), the hydrodynamics of the impinging flow in the confined impinging jet reactor under the impact of the ultrasound acoustic streaming irradiation can be modelled. Multiplying Equation (3) by the mean velocity Uj, the kinetic energy equation of the mean flow in the UTISR is expressed as:

(6) $$\frac{\partial \left(\rho \frac{1}{2}U_{i}U_{i}\right)}{\partial t}+U_{j}\frac{\partial \left(\rho \frac{1}{2}U_{i}U_{i}\right)}{\partial x_{j}}=-\frac{\partial PU_{j}}{\partial x_{j}}+\frac{\partial }{\partial x_{j}}\left[2\mu {U_{j}S}_{\mathrm{ij}}-\rho \overset{¯}{u_{i}^{\prime }u_{j}^{\prime }}U_{j}\right]-2\mu S_{\mathrm{ij}}S_{\mathrm{ij}}+\rho \overset{¯}{u_{i}^{\prime }u_{j}^{\prime }}S_{\mathrm{ij}}+U_{j}\frac{\partial \overset{¯}{p_{\mathrm{up}}}}{\partial x_{j}}$$

Similarly, the turbulent kinetic energy of the fluctuating flow can be obtained by subtracting Equation (6) from the instantaneous kinetic energy equation, which yields

(7) $$\overset{¯}{\frac{\partial \left(\rho \frac{1}{2}u_{i}^{\prime 2}\right)}{\partial t}}+U_{j}\overset{¯}{\frac{\partial \left(\rho \frac{1}{2}u_{i}^{\prime 2}\right)}{\partial x_{j}}}=-\frac{\partial }{\partial x_{j}}\left(\overset{¯}{p^{\prime }u_{j}^{\prime }}\right)+\frac{\partial }{\partial x_{j}}\left[2\mu \overset{¯}{u_{j}^{\prime }s_{\mathrm{ij}}^{\prime }}-\rho \frac{1}{2}\overset{¯}{u_{i}^{\prime }{uu_{i}^{\prime }}_{j}^{\prime }}\right]-2\mu \overset{¯}{s_{\mathrm{ij}}^{\prime }s_{\mathrm{ij}}^{\prime }}-\rho \overset{¯}{u_{i}^{\prime }u_{j}^{\prime }}S_{\mathrm{ij}}+\overset{¯}{u_{j}^{\prime }\frac{\partial p_{\mathrm{up}}}{\partial x_{j}}}$$

The last term in Equation (7) indicates the influence of ultrasound irradiation intensification on the turbulent kinetic energy generated by ultrasound irradiation. In most of the ultrasound irradiation applications, the acoustic energy absorbed by the liquid bulk is converted to the local turbulence intensification, generating very small size turbulent eddies. As the standard k-ε turbulence model has been widely used to model the turbulent flows in a confined impinging jet reactor [1], [19], [29]. Therefore, the turbulent characteristics in the UTISR and the ultrasound irradiation impact were taken into account. The modified k-ε model thus can be written as:

(8) $$\frac{\partial \left(\rho k\right)}{\partial t}+\nabla \cdot \left(\rho Uk\right)=\nabla \cdot \left(\frac{\mu_{t}}{\sigma_{k}}\nabla k\right)+2\mu_{t}S_{\mathrm{ij}}S_{\mathrm{ij}}-\rho \varepsilon +C_{\mathrm{ultra}}\overset{¯}{u_{j}^{\prime }\frac{\partial p_{\mathrm{up}}}{\partial x_{j}}}$$

(9) $$\frac{\partial \left(\rho \varepsilon \right)}{\partial t}+\nabla \cdot \left(\rho U\varepsilon \right)=\nabla \cdot \left(\frac{\mu_{t}}{\sigma_{\varepsilon }}\nabla \varepsilon \right)+C_{1\varepsilon }\frac{\varepsilon }{k}2\mu_{t}S_{\mathrm{ij}}S_{\mathrm{ij}}-C_{2\varepsilon }\rho \varepsilon \frac{\varepsilon }{k}+C_{1\varepsilon }C_{\mathrm{ultra}}\frac{\varepsilon }{k}\overset{¯}{u_{j}^{\prime }\frac{\partial p_{\mathrm{up}}}{\partial x_{j}}}$$

where $C_{\mathrm{ultra}}$ is an empirical parameter. The coefficients used in the modified k-ε model still take the following values: $C_{\mu }$ = 0.09,$\;C_{1}$ = 1.42, $C_{2}$= 1.92, $\sigma_{k}$ = 1.0 and $\sigma_{\varepsilon }$ = 1.3. The source term in Equations (8) and (9) can be expressed as

(10) $$\overset{¯}{u_{j}^{\prime }\frac{\partial p_{\mathrm{up}}}{\partial x_{j}}}\approx \lim_{T\to \infty }\frac{1}{T}\int_{t_{0}}^{t_{0}+T}u_{j}^{\prime }\frac{\partial p_{\mathrm{up}}}{\partial x_{j}}dt=\lim_{n\to \infty }\frac{1}{n\Delta t}\sum_{j=1}^{n}u_{j}^{\prime }\frac{\partial p_{\mathrm{up}}}{\partial x_{j}}\Delta t$$

The simulation time T should be long enough to ensure that the value of Equation (10) can oscillate around a certain value. Meanwhile the time step Δtshould be smaller than a quarter of the ultrasound irradiation period. Considering the chemical reaction, the species transport equations as shown in Equation (11) are solved,

(11) $$\frac{\partial }{\partial t}\left(\rho c_{i}\right)+\nabla \cdot \left(\rho Uc_{i}\right)=\nabla ∙\left[\left(\Gamma_{i}+\Gamma_{t}\right)\nabla c_{i}\right]-\frac{\partial \left(\rho \overset{¯}{u_{j}^{\prime }c_{i}^{\prime }}\right)}{\partial x_{j}}$$

where ci represents the concentration of species i, Γi is the diffusion coefficient of the species i and Γt is the turbulent diffusion coefficient. For the cases of TISR and UTISR investigated in the present study, as the density and viscosity of Fe(NO3)3 and (NH4)2HPO4 solutions are different, the Reynolds number used to characterise the mixing in the TISR can be defined by

(12) ReTISR=4ρms∑i=12Qiμmsπd

where the density ρms and viscosity μms of the mixed solution take 1.144 × 103 kg/m3 and 1.005 × 10–3 Pa·s, respectively. Qi is the feed flow rate in the inlet of the T-shape junction and di is the hydraulic diameter of the circular tube inlet. According to the experimental conditions shown in Table 4, the estimated Reynolds number falls into the range of 230–3500. However, it should be point out that it may be inappropriate to consider the flow to be still laminar as the intensive mixing taking place in the chamber where the two confined impinge jets collide to generate the turbulence and the ultrasound irradiation also induces the turbulence. Thus, the flow after impinging will likely become highly turbulent. The imposition of ultrasound irradiation will further enhance the local turbulence as the consequence of micro-cavitated bubble collapse in an extremely short time and in a very small confined volume.

**Table 4:** N_2_ adsorption–desorption analysis results of FP precursors prepared by TISR with different feeding rates.

**Sample**	Feeding rate (mL/min)	Overall Re (*Re_TISR_*)	Mean Residence Time (*t_R_*, ms)	Surface area (m^2^/g)	Total pore adsorption volume (cm^3^/g)	Average primary particle size (nm)
**S1**	17.15	233	770	21.94	0.087	273.48
**S2**	34.30	466	385	29.82	0.219	201.24
**S3**	51.44	699	257	34.75	0.263	172.69
**S4**	68.59	932	192	47.96	0.263	125.11
**S5**	85.74	1165	154	84.19	0.463	79.80
**S6**	128.61	1748	103	78.94	0.398	76.01
**S7**	171.48	2331	77	65.06	0.278	92.22
**S8**	214.35	2913	62	75.14	0.371	79.86
**S9**	257.22	3496	51	75.11	0.352	79.88

### 3.2 Numerical simulation

The geometry and computational domain are schematically shown in Fig. 2 and CFD simulation was conducted using the commercial code FLUENT 15.0. The computation domain contains a T-shaped impinging jet reactor with an ultrasonic transducer installed in the downstream sudden expansion chamber. The inner diameters of the two inlet tubes and the outlet of the reactor are 3 mm and 8 mm, respectively. The ultrasound irradiation was generated and propagated from the tip of the ultrasonic transducer which has the length of 60 mm and the diameter of 13 mm. To set up the boundary condition apropos of inlet pressure, the tip of the ultrasound probe was assumed as origin of the propagation waves, which can be clearly seen in Fig. 2, 18 mm away from the bottom of the sudden expansion chamber. The computational mesh was created using ANSYS ICEM and had an orthogonal grid feature. The preliminary simulations were performed to confirm there were no significant changes in the time-averaged concentration distributions for both the TISR and UTISR cases when the number of meshes exceeded 160,000. Therefore, this mesh setup was utilised for all subsequent simulations in this study.

The hydrodynamics of both the TISR and UTISR were modelled using CFD. The coupling between pressure and velocity was accomplished using the SIMPLE algorithm with a second-order upwind discretisation scheme, and a standard k-ɛ model was used for mixing in the reactor core due to high turbulence [15], despite the calculated Reynolds number at the reactor inlet being <2000. Velocity inlet boundary conditions were applied to both the TISR and UTISR inlets, while the pressure outlet boundary conditions were specified for the outlets of both reactors where the product was collected. The pressure inlet was set at the tip of the ultrasonic transducer. All walls were subjected to a no-slip boundary condition. Numerical simulations were performed with and without ultrasonic exposure, and ultrasound of varying amplitudes was applied with a fixed frequency of 20 kHz. The simulations were considered converged when the normalized residuals of all variables were <1 × 10–8, and the maximum number of iterations per time step was set to 100 to ensure convergence. To account for the effect of ultrasound on the bulk flow, the pressure change was described using Equation (13):

(13) $$p_{\mathrm{up}}=p_{a}cos\left(2\pi f\left(t-\frac{z}{c}\right)\right)$$

where pa is the amplitude of the ultrasound pressure (Pa), f is the frequency (Hz), t is the time (s), z is the distance measured from the tip of the ultrasound inducer and c is the local sound speed. The value of pa can be estimated based on

(14) $$p_{a}=\sqrt{2I\rho c}I=\frac{p_{\mathrm{us}}}{A}$$

## 4 Results and discussion

### 4.1 Effect of volumetric feeding rate

**Fig. 3:**
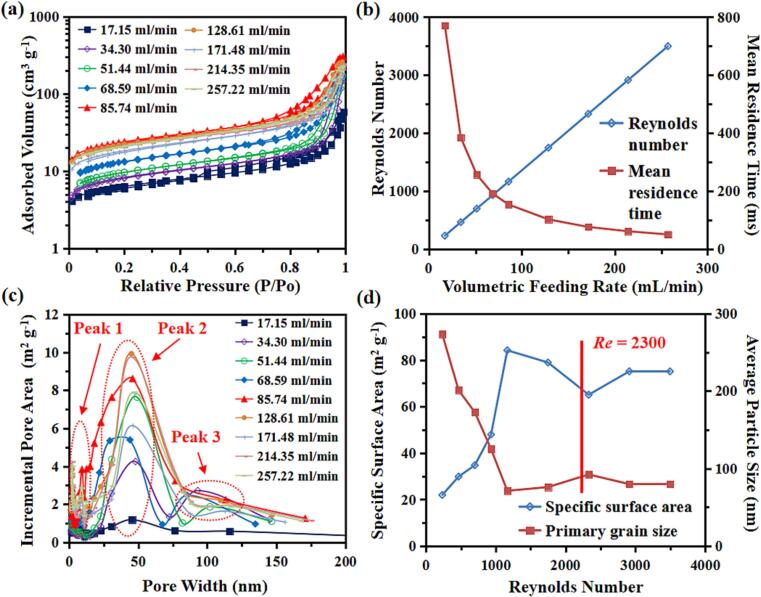
(a) N_2_ adsorption–desorption isotherms of FP samples prepared by TISR with different feeding rate; (b) the corresponding pore-size distribution obtained from the adsorption branch using the BJH method of FP samples; (c) the relationships among volumetric feeding rate, Reynolds number and mean residence time; (d) the relationships among Reynolds number, specific surface area and primary particle size.

To investigate the effect of the volumetric feeding rate, FP samples were prepared at different volumetric flow rates ranging from 17.15 to 257.22 mL/min. Table 4 and Fig. 3 show the BET analysis results of the FP precursor samples prepared with 9 different volumetric feeding rates. The N2 sorption isotherm of all samples was Type IV (as shown in Fig. 3a). When the volumetric feeding rate is relatively small (Q < 85.74 mL/min), three peaks were observed to take place in the incremental pore area as shown Fig. 3b. An increasing of volumetric feeding rate Q results in an increase in the values of Reynolds Number and a decrease in the mean residence time tR, as can be seen from Fig. 3c. As shown in Fig. 3d, when Re < 1165 based on the feeding rates, the average primary particle size of synthesised FP samples decreases remarkably with the increase in the Reynolds number and decrease in tR. In contrast to the particle size, the specific surface area increases with increase in the Reynolds number and decrease in tR. It is noted that the minimum average primary particle size (79.8 ± 8.5 nm), maximum specific surface area (84.19 m2/g) and total adsorption volume (0.463 cm3/g) were obtained when the volumetric feeding rate reaches 85.74 mL/min (Re = 1165, tR = 154 ms). The adsorbed volume of a porous particle in nitrogen (N2) adsorption–desorption analysis is used to characterize the porosity and surface area of the material. Also, the FP sample S3 has presented a porous structure that has a surface area falling into the mid of the range for all the samples. This is likely attributed to the reduced micromixing time and increased mass transfer rate due to the enhanced collision rate among the turbulent eddies, promoting the nucleation. However, further increasing the volumetric feeding rate (Q > 85.74 mL/min, corresponding to Re > 1165), the noticeable changes on the primary particle size can be observed, the size being reduced while the specific surface area being increased.

To explain this observed phenomenon, a scaling model is used to estimate the characteristic time tM for micromixing, which can be interpreted as the time for the diffusion to take place across a turbulence eddy slab with the thickness equal to the Kolmogorov length λ [10], [40],

(15) $$t_{M}=\frac{{\left(0.5\lambda \right)}^{2}}{D_{\mathrm{eddy}}}$$

where Deddy is the eddy diffusivity of the reactant solutions. The Kolmogorov length λ can be estimated based on the micromixing turbulent energy dissipation rate ε [J/s·kg] and the kinematic viscosity of the mixed solution, μms, at the point of mixing, given by

(16) $$\lambda ={\left(\frac{\mu_{\mathrm{ms}}^{3}}{\varepsilon }\right)}^{1/4}$$

where μms is the reactant mixture viscosity and can be estimated using the following expression:

(17) $$\mu_{\mathrm{ms}}=\alpha \mu_{1}+\left(1-\alpha \right)\mu_{2}=\frac{Q_{1}\mu_{1}+Q_{2}\mu_{2}}{Q_{1}+Q_{2}}$$

here, α=Q1/(Q1+Q2) is defined as the ratio of the volumetric flow rate at inlet 1 to the total volumetric flow rates. The turbulent energy dissipation should be equal to the energy change in the impinging stream reactor. Thus, the volumetric averaged turbulent energy dissipation rate can be estimated by the ratio of the input power P (or the product of the pressure drop to the volumetric flow rate across the reactor) to the mixed solution mass in the reactor. For the configuration of the TISR used in this work, this can be expressed by Equation (18):

(18) $$\langle \varepsilon \rangle =\frac{P}{\rho_{\mathrm{ms}}V_{\mathrm{TISR}}}$$

where ρms is the mixed solution density, estimated by:

(19) $$\rho_{\mathrm{ms}}=\alpha \rho_{1}+\left(1-\alpha \right)\rho_{2}=\frac{Q_{1}\rho_{1}+Q_{2}\rho_{2}}{Q_{1}+Q_{2}}$$

and VTISR is the mixing volume of the impinging streams in the reactor. The impinging streams have been assumed to be incompressible and the flow rate at the outlet is equal to the sum of the flow rates of two inlets. ρ1 and ρ2 are the densities of the impinging stream solutions at inlets 1 and 2. The kinetic energy losses in the reactor can be approximately considered to be equal to the pressure drop taking place in the TISR. As both the two inlets and outlet of the T-junction have the same cross-sectional area, the total kinetic energy loss caused by the impinging streams in the TISR, i.e. the pressure drop across the reactor, can be estimated by

(20) $$\Delta p=\xi_{\mathrm{TISR}}\frac{U_{3}^{2}}{2}=\xi_{\mathrm{TISR}}\frac{{8Q}_{3}^{2}}{\pi^{2}d^{2}}=\xi_{\mathrm{TISR}}\frac{8{\left(Q_{1}+Q_{2}\right)}^{2}}{\pi^{2}d^{2}}$$

where ξTISR is the local loss coefficient which can be estimated using the following empirical relation

(21) $$\xi_{\mathrm{TISR}}=1+\varphi^{2}+3\varphi^{2}\left(q_{\mathrm{ISR}}^{2}-q_{\mathrm{ISR}}\right)$$

where qTISR = Q1/(Q1 + Q2) is the discharge ratio and φ = A1/A3 the ratio of the cross-sectional areas of the inlet 1 to outlet. For the experimental condition used in the current study, qTISR is taken to be 0.5 while φ takes 1 and ξTISR takes 1.25. The pressure drop across the reactor can thus be estimated by

(22) $$\Delta p=\xi_{\mathrm{TISR}}\frac{8{\left(Q_{1}+Q_{2}\right)}^{2}}{\pi^{2}d^{2}}=1.25\frac{64Q^{3}}{\pi^{2}d^{2}}.$$

When assuming the turbulent energy dissipation taking place in the reactor to be mainly contributed by the pressure drop across the TISR, then the volumetric averaged turbulent energy dissipation rate ɛ and the Kolmogorov length λ in the TISR, when substituting Equation (22) into (18), are thus estimated, respectively, by

(23) $$\langle \varepsilon \rangle =\frac{1.25}{\rho_{\mathrm{ms}}V_{\mathrm{TISR}}}\frac{64Q^{3}}{\pi^{2}d^{2}}$$

(24) $$\langle \lambda \rangle ={\left(\frac{\mu_{\mathrm{ms}}^{3}\rho_{\mathrm{ms}}V_{\mathrm{TISR}}\pi^{2}d^{2}}{80Q^{3}}\right)}^{1/4}.$$

Thus, the micromixing time tM can be estimated using Equation (25):

(25) $$t_{M}=\frac{{\left(0.5\langle \lambda \rangle \right)}^{2}}{D_{\mathrm{eddy}}}=\frac{0.25}{D_{\mathrm{eddy}}}{\left(\frac{\mu_{\mathrm{ms}}^{3}\rho_{\mathrm{ms}}V_{\mathrm{TISR}}\pi^{2}d^{2}}{80Q^{3}}\right)}^{1/2}.$$

The increase in the volumetric feeding rate Q leads to the enhancement of the mass transfer due to intensified diffusion through the engulfment of two solutions among the Kolmogorov length scale eddies and improved collision rate. In addition, the turbulent energy dissipation rate ɛ will increase as the result of increasing volumetric feeding rates. It should be pointed out here that the Kolmogorov length λ would decrease with increasing Reynolds number such that the micromixing time tM is decreased. As high level of supersaturation can be achieved in TISR, homogeneous nucleation will occur and large amount of FP nucleus are generated. The heterogeneous nucleation will then start due to the presence of FP nucleus and reduced supersaturation level of solution. When these FP nuclei are transported with turbulent eddies, the local turbulent shear stresses acting on the FP nuclei may be smaller than the bonding forces among these trapped FP nuclei, the FP particles start to grow in size. When the volumetric feeding rate is lower than 85.74 mL/min (Re < 1165), an increase in the feeding velocity may give rise to an increased mass transfer rate and nucleation rate, a shortened mean residence time tR and micromixing time tM. This is helpful for achieving a smaller primary particle size, larger specific surface area and a higher adsorption pore volume. When further increasing the volumetric flow rate to exceed 85.74 mL/min, the average primary particle size and specific surface area are less responsive to the volumetric feeding rate. This may be attributed to a balance between the local turbulent shear stress acting on the particles and the other forces due to the chemical reaction. Under this condition, the Da number, or the ratio of the micromixing homogenization time (tM) and nucleation time (tN) should be in the order of 1. Due to the same initial reagent concentration and temperature, the chemical reaction rate and tN were maintained at the same level. Further increasing in the volumetric feeding velocity can enhance mass transfer while reduce the micromixing homogenization time tM and mean residence time within the mixing zone of TISR (tR). Thus, the desirable mixing of reactant can be achieved through shortening tN.

**Fig. 4:**
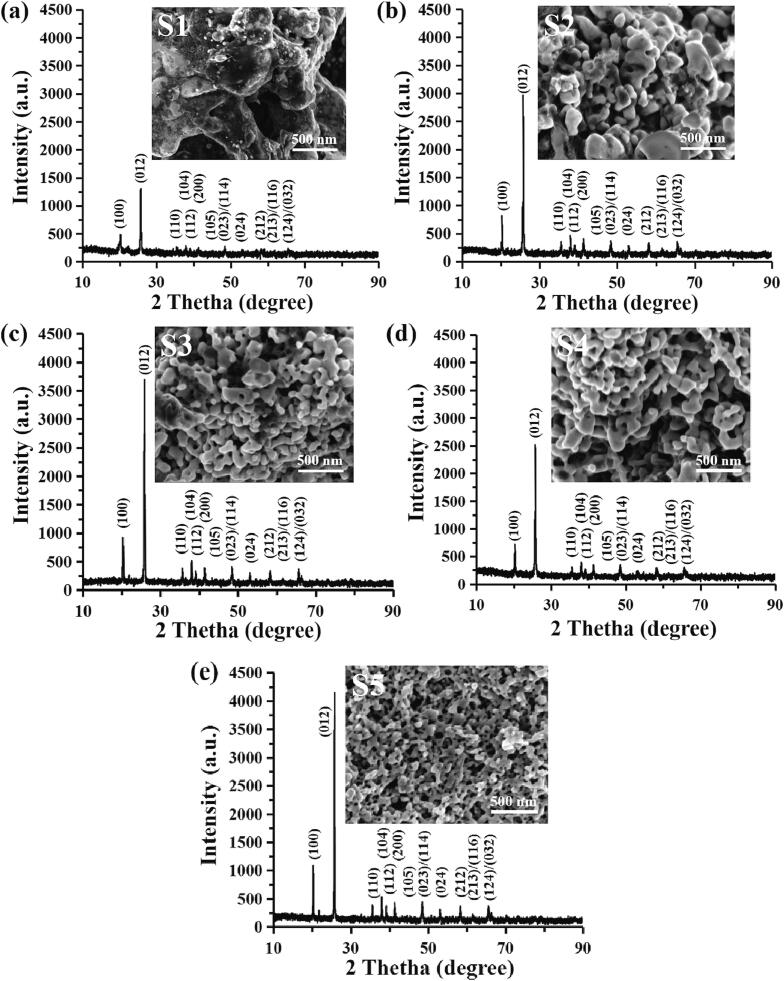
X-ray diffraction patterns and SEM images of FP samples synthesized at different volumetric feeding rates: (a) S1; (b) S2; (c) S3; (d) S4 and (e) S5.

Fig. 4 shows significant changes in the XRD results and SEM images when the FP samples were prepared by increasing volumetric feeding rate (Q) from 17.15 to 85.74 mL/min (feeding velocity range from 0.040 to 0.202 m/s) and being calcined at 600 °C for 10 h. All the XRD patterns of FP fit the ideally crystallized hexagonal structure FP (JCPDS card no. 29–0715, a = 5.035 Å, b = 5.035 Å, c = 11.245 Å). The diffraction peak at 2θ = 20.3o and 25.8o becomes higher and sharper as the volumetric flow rate is increased, indicating that the use of higher volumetric flow rate is able to produce FP particles with smaller particle size. As expected, the SEM analysis (Fig. 4) indicates that an increase in the volumetric flow rate can result in an increase of porosity and a reduction of particle size of as-prepared FP samples while smaller particle size will have bigger morphological surface area. At low volumetric flow rate (Q = 17.15 mL/min), the as-prepared FP sample S1 (Fig. 4a) shows irregular particle shape with typical diameter over 400 nm. Meanwhile, the specific surface area of S1 is only 21.94 m2/g (Table 4). When the volumetric feeding rate is increased to 34.30 mL/min, the particle size of S2 reduces to 201.24 nm, while the surface area increases to 29.82 m2/g. As shown in Fig. 4c, it is interesting to see that a more mesoporous surface can be observed. The particle size and surface area of S3 are 172.69 nm and 34.75 m2/g, respectively. Although the particle size (125.11 nm) and surface area (47.96 m2/g) of S4 are different with S3, these two samples reveal similar total pore adsorption volume (0.263 cm3/g). As the volumetric flow rate is increased to 85.74 mL/min, the particle size of FP decreases to 50–100 nm, showing a similar trend with BET results (79.80 nm). Moreover, based on XRD results, the calculated mean crystalline size of these samples are 203.3 nm (S1), 169.1 nm (S2), 126.7 nm (S3), 87.8 nm (S4) and 64.1 nm (S5), which shows a similar tendency with BET results and SEM images.

The finding on effect of the feeding flow rate on the particle size was consistent with the research which depicts that increasing the liquid flow rate in a coaxial jet processor led to a decrease in the mean particle size and an increase in the particle size distribution [41]. Specifically, the study found that liquid flow rate doubles at a relatively low flow rate from 10 to 20 mL/min resulting in a decrease to 40% in the mean particle size from 470 to 280 μm. Comparatively, the particle size decreased to 26.4% in our experiment from 273.48 nm to 201.24 nm when the feeding rate is doubled from 17.15 mL/min to 34.30 mL/min. However, this may cause a relative increase in the spread of particle sizes. The particle size distribution increases from 0.8 to 1.1, representing a relative increase in the spread of particle sizes. This is also verified by the research on the effect of liquid jet flow rate on particle size distribution in a coaxial atomization spray dryer [42]. The study found that increasing the liquid flow rate led to a decrease in the mean particle size and an increase in the particle size distribution with an increase in the particle size distribution from 0.3 to 0.6.

**Fig. 5:**
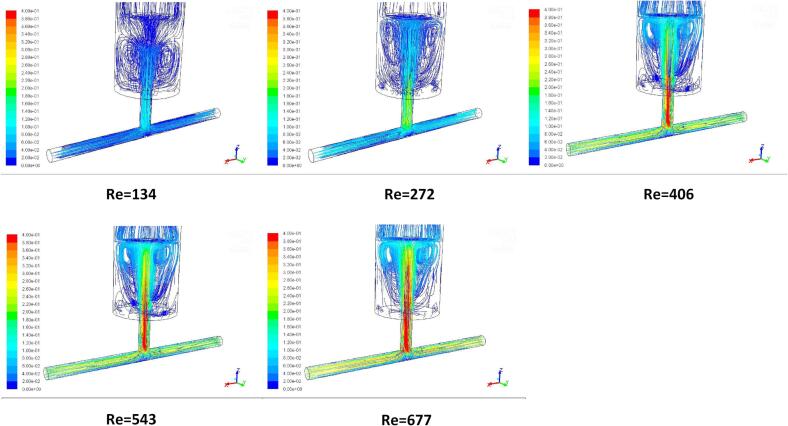
Pathline patterns of the impinge flows in cutting plane of the T-junction impinge jet flow reactor for various Reynolds number at the impinge flow time *t* = 1.0 s without adopting ultrasound irradiation.

The tracking of fluid particles was conducted using CFD simulations of fluid particle trajectories in the impinging jet reactor as shown in the Fig. 5. The pathlines obtained for the case of Re = 134 clearly shows the radial spread of the vortices surrounding the central axis downstream the outlet of impinging jets after two opposite jet flows impinge each other from the two cylindrical tubes into the impinge chamber. When Re is increased to 272, an irregular pattern of recirculation vortices is formed in the vicinity of the tip of the ultrasound transducer. At the same time, the turbulent kinetic energy generated increases with an increase in the Reynolds as can be seen clearly from Fig. 5. Such deformed large eddies intensifies the generation of the local turbulent energy dissipate rates so that the local turbulent shear stresses may confine the particle nuclei growth. Consequently, the aggregated particle size can be controlled.

**Fig. 6:**
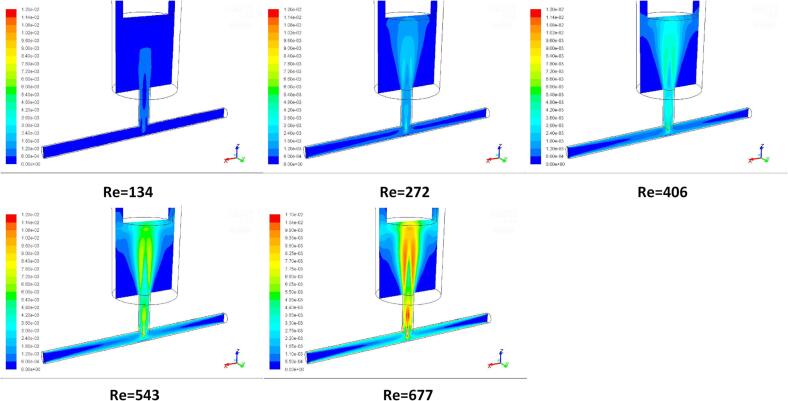
Contours of turbulent kinetic energy (k) at intersecting surface of TISR versus Reynolds number Re reactor at the flow time *t* = 1.0 s without ultrasound irradiation.

**Fig. 7:**
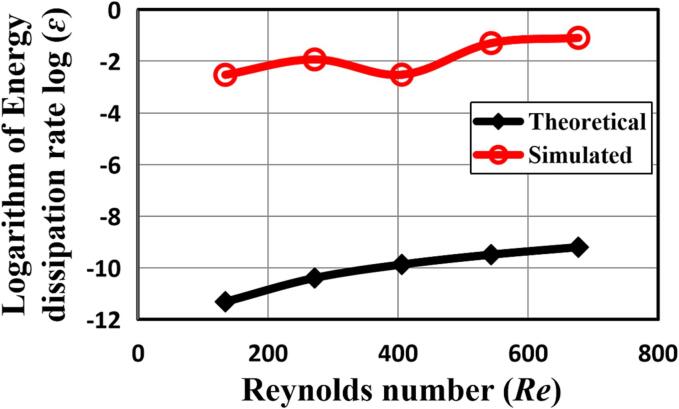
Logarithm of energy dissipation rate ε versus Reynolds number *Re* in the impinging jet reactor at the flow time *t* = 1.0 s without ultrasound irradiation.

Fig. 6 shows the contours of turbulent kinetic energy k corresponding to Fig. 5 for various Reynolds numbers at the impinging flow time t = 1.0 s. Comparing the results of the turbulent kinetic energy dissipation rates with the pathline patterns, it can be clearly seen from both figures that with an increase in the Reynolds number, the turbulent kinetic energy generation becomes apparent. This gives rise to the increase in the local turbulent energy dissipation rates. Fig. 7 shows the comparisons of the estimated turbulent energy dissipation rate 〈ε〉 based on Equation (23) with the calculated volumetric average ε obtained by CFD simulation. Note that a logarithmic scale has been used in the figure to capture the wide range of the impinge flow rates for estimation of ε. As the turbulent kinetic energy is enhanced with increase in the Re, the corresponding turbulent energy dissipation rate would increase, which would generate smaller turbulent eddies. Consequently, the particles nucleation and growth in the synthesis process would be engulfed by these very small eddies and experienced relatively strong local turbulence induced shear stress.

To further explore the impact of adoption of different volumetric feeding rate, i.e. the use of different Reynolds number, on the micromixing behaviour in the impinging jet reactor, especially for the zone just downstream of the impinge jet, a special correlation between the cross-section area-averaged turbulent kinetic energy k and the local concentration of the products ci was proposed. $R_{k,c_{i}}$ represents the correlation factor and it can be calculated using the modelling results obtained from Equation (11), given by

(26) $$R_{k,c_{i}}=\frac{\overset{¯}{kc_{i}}}{\sqrt{\overset{¯}{k^{2}}\sqrt{\overset{¯}{c_{i}^{2}}}}}.$$

**Fig. 8:**
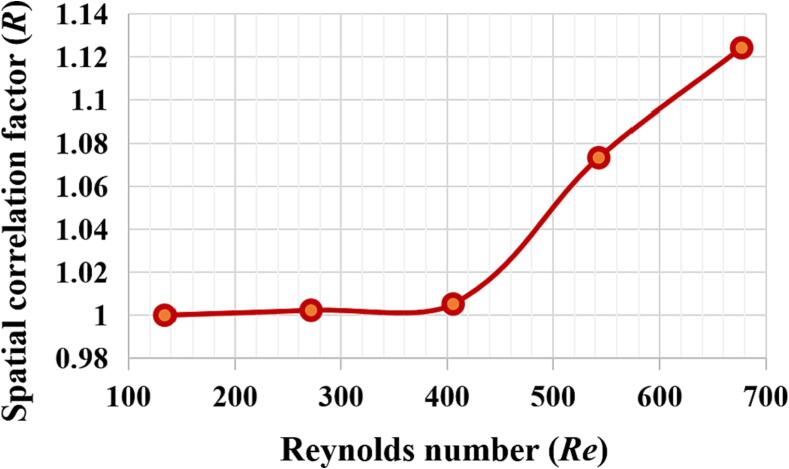
Spatial correlation versus Reynolds number Re in the mixing chamber at the flow time *t* = 1.0 s without ultrasound irradiation.

The results of such correlation are shown in Fig. 8. It can be seen from the figure that the correlation $R_{k,c_{i}}$ presents the trend of increasing with an increase in the Reynolds number especially when the Reynolds number is greater than 400. This may be attributed to the fact that when the feeding rate increases, the turbulent energy dissipation rate also increases, giving rise to an enhanced local turbulent induced shear. The increase in the correlation factor likely indicates the increased contribution of the turbulence to the local micromixing such that the properties of the synthesized FP particles are affected. Thus, the use of volumetric feeding rate has an import impact on the synthesis of FP particle size.

### 4.2 Effect of the power of ultrasound irradiation

The influence of ultrasound intensity was evaluated when FP samples were prepared at different ultrasound irradiation powers ranging from 0 W to 900 W. With the help of ultrasound irradiation, the sonolysis of the water can be characterised by the following reactions [37]:

H2O → H·+OH·

H·+H·→H2

OH·+OH·→H2O2

H·+OH·→H2O.

For the preparation of FP particles, the main and side reactions are as follows:

Fe3++PO43-→FePO4↓

Fe3++3OH–→Fe(OH)3↓

In this work, the synthesis of FePO4 and Fe(OH)3 is a homogeneous competitive-consecutive (series–parallel) reaction. In order to facilitate the main reaction, the mixing intensity should be enhanced via the impinge jets to increase the ion collision between Fe3+ and PO43−. Although the solubility product constant of Fe(OH)3 (Ksp = 2.8 × 10–38) is much lower than FePO4 (Ksp = 1.3 × 10–22), the side reaction could be ignored as the reaction circumstance is acidic.

**Fig. 9:**
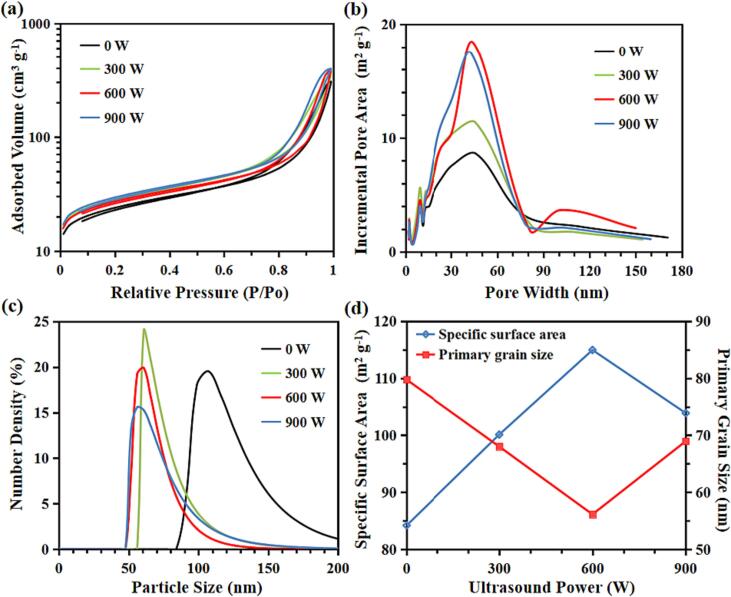
(a) N_2_ adsorption–desorption isotherms; (b) pore-size distribution; (c) particle size distribution (evaluated by DLS); (d) the relationships among ultrasound power, specific surface area and average primary particle size.

The BET results of the as-synthesised particles were characterized by applying the N2 adsorption–desorption analysis. The N2 sorption isotherm of all FP particles (Fig. 9a) was Type IV [30]. Their big hysteresis loops have provided the evidence for the presence of mesopores (between 2 and 50 nm) and large surface area. In addition, Fig. 9b shows that all the FP samples synthesised at different ultrasound irradiation intensity are feature pores in mesoporous (between 2 and 50 nm) and small-macroporous (between 50 and 85 nm) range. Moreover, increasing in ultrasound irradiation power leads to the changing of average nuclei size, porosity, and surface area of FP samples, which is shown in Table 5 and Fig. 9b-c. The average particle size was determined based on the results of BET and DLS. A significant decline of average primary particle size (from 79.8 ± 8.5 nm to 56.1 ± 10.5 nm), and increase in both total pore adsorption volume (from 0.463 cm3·g^−1^ to 0.570 cm3·g^−1^) and specific surface area (from 84.19 m2·g^−1^ to 114.97 m2·g^−1^) can be obtained with increasing ultrasound irradiation power from 0 W to 600 W. However, it was found that when increasing the ultrasound irradiation power to a value higher than 600 W, increases in the average primary particle size and a reduction in the specific surface area were observed.

**Table 5:** Specific surface area, total pore adsorption volume, and average primary particle size of FP samples synthesised with different ultrasound power.

Samples	Specific surface area (m^2^·g^−1^)	Total pore adsorption volume (cm^3^·g^−1^)	Average primary particle size (nm)	Ultrasound power (W)
S5	84.19	0.463	79.80	0
S10	100.12	0.524	60.78	300
S11	114.97	0.570	56.10	600
S12	103.89	0.608	62.89	900

**Fig. 10:**
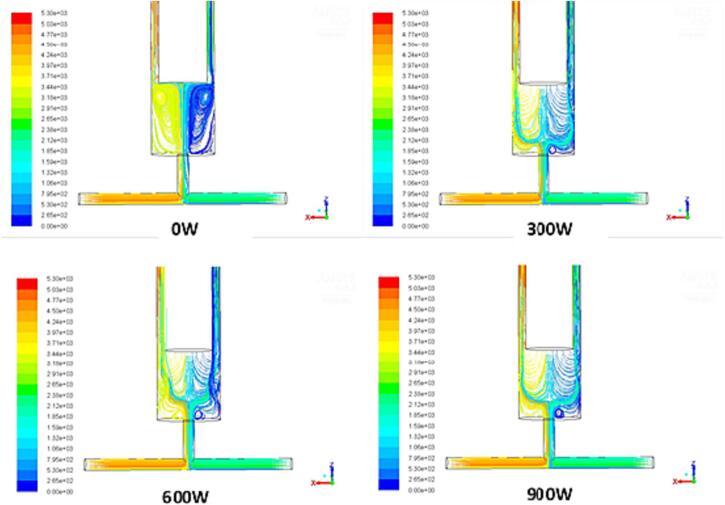
Pathlines of flow at intersecting surface of impinging jet reactor versus ultrasound intensity at the inlet velocity *v* = 0.202 m/s and flow time *t* = 1.0 s.

**Fig. 11:**
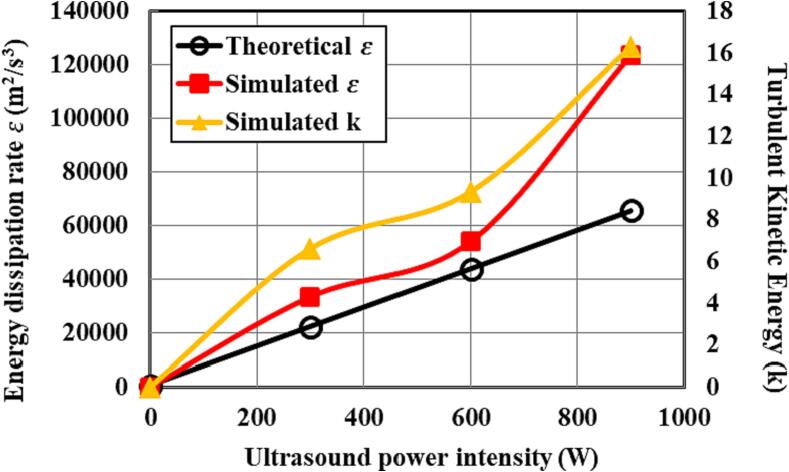
Energy dissipation rate versus ultrasound power intensity in the impinging jet reactor at the inlet velocity *v* = 0.202 m/s and flow time *t* = 1.0 s.

The results can be explained with the aid of CFD simulations. Fig. 10 shows the pathlines of the flow at the intersecting surface of mixing chamber under different ultrasound intensity at the constant inlet velocity of 0.202 m/s. With the adoption of ultrasound irradiation, the vortices formed below the ultrasound inducer change their positions. However, without imposing the ultrasound irradiation, two opposed jets entered the chamber in the normal direction to the jets’ axes and then two symmetric vortices formed near the tip of the ultrasound transducer. With imposition of the ultrasound irradiation, the vortices are deformed at the tip of the ultrasound transducer and are forced to move downstream, closing to the outlet of the expansion chamber of the impinge jet. It can be seen from Fig. 11 that the volumetric average turbulent kinetic energy and turbulent energy dissipation rate are enhanced as the result of imposition of the ultrasound irradiation, which would intensify the local turbulence induced shear and generate more small turbulent eddies such that the particle size can be further reduced.

As the same volume of the reaction chamber of T-junction and volumetric feeding rates were adopted for both the UTISR and TISR, the mean residence time of the samples synthesized with different ultrasound irradiation intensity was kept the same. However, the ultrasound irradiation intensity would affect the micromixing time and chemical reaction rate significantly. A scaling model was used to estimate the characteristic micromixing time tM, which can be expressed as

(27) $$t_{M}=\frac{[0.5{{\left(\frac{\rho_{\mathrm{ms}}V_{T}v_{\mathrm{ms}}^{3}}{P_{\mathrm{IS}}+P_{\mathrm{UI}}}\right)}^{1/4}]}^{2}}{D_{\mathrm{eddy}}}.$$

In addition, Ratoarinoro et al. (1995) proposed the evaluation of the overall reaction rate r0 under sonication using the following relationship [32]:

(28) $$r_{o}=k_{l}C_{o}\frac{6m_{p}}{d_{32}\rho_{p}V_{l}}$$

where $k_{l}$ is mass transfer coefficient, $C_{o}$ is reagent initial concentration, d 32 is Sauter mean diameter, which is the mean diameter over the surface distribution, $V_{l}$ is the reaction liquid volume that can be assumed to be equal to the volume of reactor chamber VT, $\rho_{p}$ and $m_{p}$ are the density and mass of the solid particles, respectively.

**Fig. 12:**
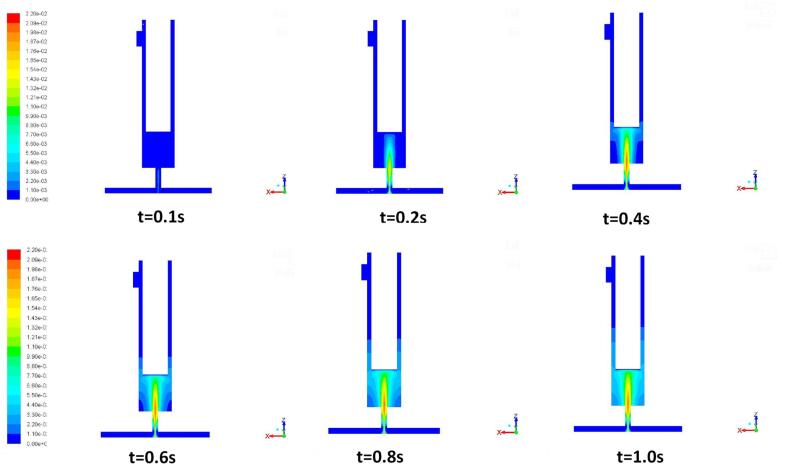
Contours of mass fraction of product FePO_4_ at different instants of time when the inlet velocity *v* = 0.202 m/s.

The theoretical results of the energy dissipation rate derived using Equation (23) are quite consistent with the volumetric average turbulent kinetic energy and turbulent energy dissipation rates using the CFD modelling as shown in Fig. 12. With the adoption of the ultrasound irradiation, the energy dissipation is subsequently greater than that without imposing the ultrasound irradiation. The averaged dissipation rate increases as the result of intensification of the ultrasound power, which the local turbulent energy dissipation rate would increase such that the stronger local turbulent shear stress can be obtained, giving rise to the synthesized particles with a smaller size.

**Fig. 13:**
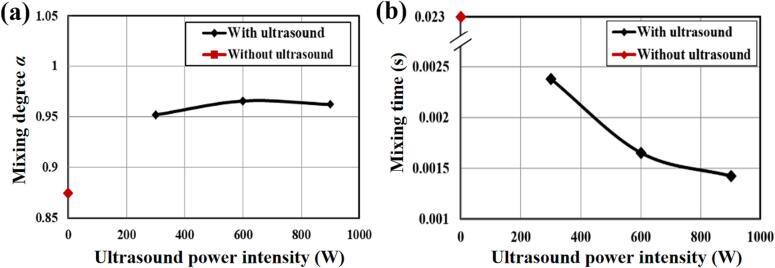
The mixing degree α (a) and mixing time (b) versus ultrasound power intensity in the impinging jet reactor (UTISR case) at the inlet velocity *v* = 0.202 m/s and flow time *t* = 1.0 s.

Fig. 13 shows the synthesized FePO4 concentration development with time, predicted by the simulation. In order to demonstrate the impact of imposition of the ultrasound irradiation on the micromixing, the mixing performance of reaction is evaluated by the mixing degree α, defined by [1]

(29) $$\alpha =1-\sqrt{\frac{\overset{¯}{c^{\prime 2}}}{\overset{¯}{c_{\max}^{\prime 2}}}}$$

where $\overset{¯}{c^{\prime 2}}$ is the mean square of normalised concentration of synthesised particles and $\overset{¯}{c_{\max}^{\prime 2}}$ is the maximum mean square of normalised concentration. It can be observed from Fig. 13a that the mixing degree is approximately equal to 0.87 without imposing ultrasound irradiation (case of TISR) and it has been enhanced to 0.95 when 300 W ultrasound irradiation was applied. As the ultrasound irradiation intensity increases, the mixing degree approaches a constant. This can be explained by the fact that the size of the smaller turbulent eddies may not change very much for further increasing the ultrasound irradiation power as those micro cavitated bubbles induced by ultrasound will have less change on their collapse size. The impact on the micromixing performance by imposing ultrasound irradiation can be also assessed by estimation of the micromixing time tm, based on [2],

(29) $$\langle t_{m}\rangle =K\sqrt{\frac{\nu }{\langle \varepsilon \rangle }}$$

where K is the factor, ν is the kinematic viscosity and the ε is the volumetric average specific energy dissipation rate. The micromixing time tm is independent of the chemical reaction and only relies on the hydrodynamics inside the reactor. The calculated micromixing time tm with the applied ultrasound irradiation power for the reagents is shown in Fig. 13. It can be seen from the figure that as the ultrasound irradiation power increases, the micromixing time tm decreases from 0.003 s to 0.0023 s for case of applying ultrasound irradiation of 300 W. This clearly indicates that the adoption of ultrasound irradiation can noticeably reduce the micromixing time and the overall the mixing performance can be enhanced significantly.

### 4.3 Synergistic effect of volumetric flow rate and ultrasound irradiation

FP samples were also synthesised at five different levels (Q = 17.15, 34.30, 51.44, 68.59 and 85.75 mL·min-1) by the UTISR to reveal how the synergistic effect of the volumetric flow rate and ultrasound intensity affects the synthesis process and the final products properties. As shown in Fig. 3a, all the samples have shown Type IV N2 sorption isotherm, which clearly indicated the existence of mesopores and large surface area by the big hysteresis. In Fig. 3c, it can be seen clearly that the pore width of samples synthesized by TISR ranges from 10 nm to 90 nm. In contrast, the application of ultrasound irradiation can be beneficial to control the generation of pores with three different diameters, which are: (1) relatively small mesopores (5–10 nm), (2) mesopores and small macropores (20–80 nm), and (3) macropores (larger than 90 nm). The appearance of Types 1 and 2 can be attributed to the generation of intraparticle pores, which are formed with the aid of the agglomeration of primary nano-particles and cavitated bubble implosion due to the ultrasound irradiation. Meanwhile, the appearance of Type 3 can be attributed to interparticle pores, formed by the breakup of larger size particles. Generally speaking, as Q increases, the FP samples synthesised by using the UTISR present relatively uniform and larger pore area when the pore width ranges from 20 to 80 nm. While the electrode with a uniform pore size can give out the better open volume, leading to a better mass transport without occurrence of wasted space [10].

**Fig. 14:**
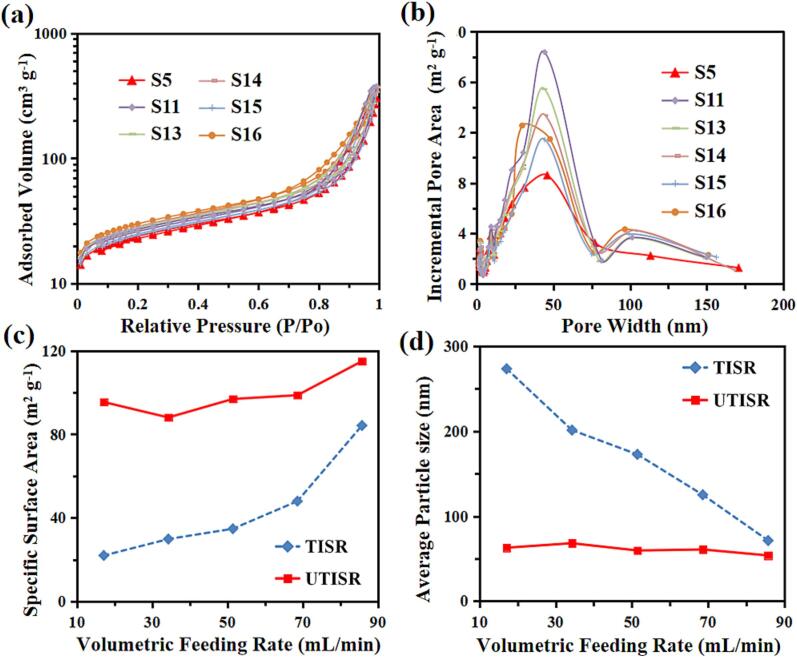
(a) N_2_ adsorption–desorption isotherms; (b) the corresponding pore-size distribution; (c-d) the relationship between Reynolds number and specific surface area and average primary gain size.

**Fig. 15:**
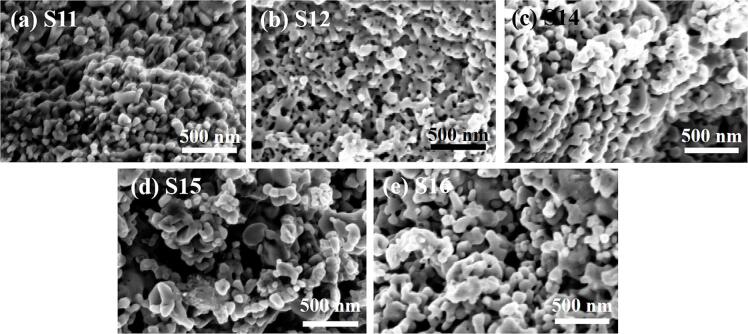
SEM images of FP samples prepared by UTISR method.

As shown in Table 6 and Fig. 14, there is a significant increase of total pore adsorption volume and specific surface area of the FP particles prepared by using the UTISR. When the volumetric feeding rate was 17.15 mL·min^−1^, the total pore adsorption volume and specific surface area of the S1 sample were found to be 0.087 cm3·g^−1^ and 21.94 m2·g^−1^, while a dramatic increase corresponding to pore adsorption volume of 0.587 cm3·g^−1^ and specific surface area of 95.41 m2·g^−1^ were obtained when ultrasound intensity is 600 W (S16), corresponding to 6.7 times and 4.3 times respectively. In Fig. 15, the SEM images reveal the FP particles prepared by UTISR exhibit smaller particle size than the FP particles prepared by TISR. Especially in Fig. 4a and Fig. 15e, when the volumetric feeding rate is maintained at 17.15 mL/min, the average primary particle size decreases significantly from 273.48 nm (S1) to 62.89 nm (S16) due to the application of ultrasound irradiation. Meanwhile, the surface area and total pore adsorption volume also increased from 21.94 m2/g and 0.087 cm3/g (S1) to 95.41 m2/g and 0.587 cm3/g (S16).

**Table 6:** BET results and Re number of FP prepared with different *Q* by UTISR methods (Reagent concentration = 1.0 mol·L^-1^).

**Sample**	Ultrasound power (W)	Feeding rate (mL/min)	Overall Re (*Re_TISR_*)	Surface area (m^2^/g)	Total pore adsorption volume (cm^3^/g)	Average primary particle size (nm)
**S11**	600	85.74	1165	114.97	0.570	56.10
**S13**		68.59	932	98.72	0.584	60.78
**S14**		51.44	699	96.88	0.576	59.60
**S15**		34.30	466	88.05	0.522	68.14
**S16**		17.15	233	95.41	0.587	62.89

However, the average primary particle size, pore adsorption volume, and specific surface area of the FP samples synthesized by UTISR change slightly with an increase in the feeding rate Q when the ultrasound irradiation power is maintained at 600 W. In Fig. 15(a-e), all the FP samples show similar morphology consisting of spherical-like particles that interweave together and form high porosity structure. For S16 (17.15 mL/min, 600 W), the average primary particle size, pore adsorption volume, and specific surface area were found to be 62.89 nm, 95.41 m2·g^−1^, and 0.587 cm3·g^−1^, respectively, gradually changing to 56.10 nm, 114.97 m2·g^−1^, and 0.570 cm3·g^−1^ when Q increases to 85.75 mL·min^−1^ and ultrasound intensity maintains at 600 W (S11).

**Fig. 16:**
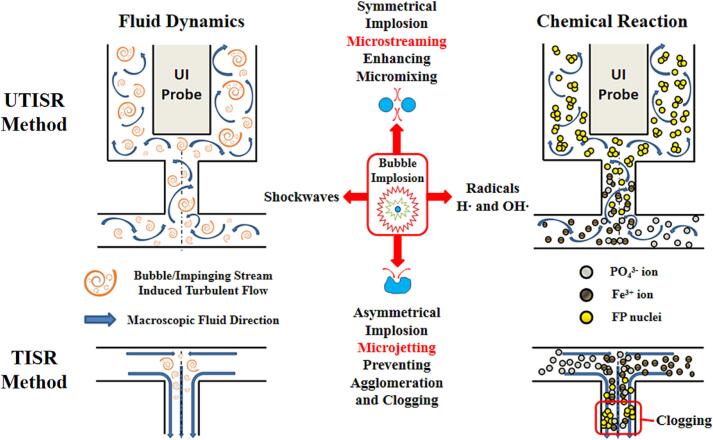
Schematic diagram of fluid dynamics and chemical reaction in UTISR and TISR system.

Based on Equations (23) to (25), it can be seen clearly that the Reynolds number, micromixing time, and energy dissipation rate in the TISR are only dependant on the volumetric feeding rate Q when all the other factors are maintained at the same level. An increase in the volumetric feeding rate can give rise up an intensified diffusion through the engulfment of two solutions among the Kolmogorov length scale small eddies, which leads to the enhanced mass transfer rate and energy dissipation rate, together with the shortened mean residence time and micromixing time. Fig. 16 shows the schematic diagram of fluid dynamics and chemical reaction in the UTISR and TISR reactors. It can be seen from the figure that the joint actions of the impinging streams and ultrasound irradiation can induce the turbulence though the impinge streams are still under high Reynolds number laminar flows. In the TISR system, turbulence only exists in the impinging zone, which is located in the center of T-type impinging microreactor (shown in Fig. 1b). While for the UTISR system, the implosion of cavitated bubbles due to the application of ultrasound irradiation can provide an instantaneous extremely high local pressure and temperature condition which would be beneficial to the synthesis reaction. This process can be regarded as the existence of many microreactors which can generate powerful hydraulic shocks. At the beginning of the experiment, as there are no particles present in the reactant solutions, the reaction taking place in the reactor can be considered as homogeneous reaction. The implosion of the microbubbles induced as a result of applying ultrasound irradiation generates micro-streaming and very small turbulent eddies, which could transfer the flow regime to local highly turbulent, leading to the generation of local high turbulent energy dissipation rate. As a result, the application of ultrasound irradiation can significantly improve the local micromixing and diffusion rate among these eddies. The synthesis process of the FP nano-seeds was significantly improved due to the enhanced nucleation rate and chemical reaction rate. After the FP nanoscale seeds are generated, the regime in the reactor becomes heterogeneous. The high local turbulence dissipation rate would generate turbulence induced shear, which can prevent from agglomeration and clogging among particles or adhesiveness between particles and the internal surface of the reactor. Thus, the enhanced micromixing effect and local mass transfer as the result of micro-engulfment are beneficial to the crystal nucleation in the process of formation of precursor particles in UTISR. This implies that the local micromixing can be significantly improved more by imposing ultrasound irradiation compared to increasing the feeding rate Q. However, the synergic effect of jointly imposing the ultrasound irradiation and increasing the feeding rate would lead to the generation of particles with smaller size, enlarged specific surface area, narrower and sharper particle size distribution.

## 5 Conclusions

In the present work, FP particles are synthesized and prepared by using the UTISR approach. The synergistic effect of ultrasound irradiation and volumetric feeding rates on synthesised particle size, porosity, specific surface area and electrochemical performance are systematically studied. The conclusions reached from the present work can be summarised as follows:

Increasing the ultrasound irradiation intensity can reduce the eddy micromixing time and the overall reaction rates. Consequently, the Da number has been reduced. As a result, a smaller primary particle size, higher specific surface area, and narrower and sharper pore size distribution can be obtained. However, an over-increase in the ultrasound irradiation intensity would have a negative impact, resulting in an increase of the average particle size, and a reduction of the specific surface area due to the extremely high temperature caused by higher ultrasound intensity.

An increased specific surface area and total adsorption pore volume, and reduced average primary particle size can be obtained by increasing the volumetric flow rates for both the TISR and UTISR used in the present study. These effects become particularly apparent when using the TISR. As the turbulent kinetic energy dissipation rate and micromixing time in the T-type micromixer are only related to the volumetric feeding rate Q, increasing the feeding flow rate would enhance the turbulent kinetic energy generation, such that an enhanced local turbulence induced shear can be generated, which could help reduce the particle pore size. However, for case of the UTISR, the implosion of microbubbles due to ultrasound irradiation can induce turbulent eddies which would promote the generation of the local turbulence. Consequently, the overall turbulent energy dissipation can be further enhanced comparing to the TISR. The micromixing effect can be significantly improved more by applying the ultrasonic irradiation than by increasing the volumetric feeding rates.

It has been affirmed that increasing the Reynolds number Re and the adopted ultrasound power leads to the reduction of Da number, indicating that the effect of turbulence induced shear on the synthesised particles increases compared with the effect of hydrolysis in the synthesis process. The adoption of ultrasound irradiation can significantly enhance the intensity of turbulence embedded in the T-junction reaction chamber and change the local turbulent energy dissipation rate as characterised by the local turbulent shear $\sqrt{\frac{\varepsilon }{\nu }}$.

## Declaration of Competing Interest

The authors declare that they have no known competing financial interests or personal relationships that could have appeared to influence the work reported in this paper.
